# Early results of laparoscopic single‑anastomosis duodeno‑ileal bypass with sleeve gastrectomy: a case series from a single Polish bariatric center

**DOI:** 10.20452/wiitm.2024.17912

**Published:** 2024-11-19

**Authors:** Mateusz Wityk, Maciej Bobowicz, Mateusz Pryt, Natalia Dowgiałło-Gornowicz

**Affiliations:** Department of General and Oncological Surgery, Regional Health Centre, Lubin, Poland; Department of Radiology, Medical University of Gdansk, Gdańsk, Poland; Department of General and Oncological Surgery, Janusz Korczak Regional Specialist Hospital, Słupsk,, Poland; Department of General, Minimally Invasive and Elderly Surgery, Collegium Medicum, University of Warmia and Mazury, Olsztyn, Poland

**Keywords:** bariatric surgery, diabetes, hypertension, metabolic surgery, obesity

## Abstract

**INTRODUCTION:**

The obesity epidemic has led to an increased prevalence of related conditions, such as type 2 diabetes (T2D) and hypertension. While laparoscopic sleeve gastrectomy is the most common metabolic bariatric surgery, up to 50% of patients may require revisional procedures due to weight regain or comorbidity recurrence. Single-anastomosis duodeno-ileal bypass with sleeve gastrectomy (SADI-S) is emerging as an effective treatment option with promising short-term outcomes.

**AIM:**

This study aimed to present the outcomes of patients who underwent SADI-S or revisional SADI.

**MATERIALS AND METHODS:**

This retrospective, single-center cohort study included 12 patients who underwent SADI-S or SADI between February 2023 and March 2024. The patients were assessed for percentage of total weight loss (%TWL), remission of T2D and hypertension, length of hospital stay, operative time, and complications. All outcomes were reported according to the American Society for Metabolic and Bariatric Surgery standards.

**RESULTS:**

A total of 9 patients underwent primary SADI-S and 3 underwent revisional SADI. The mean (SD) %TWL was 27.9% (4.3%) at 6 months and 31.1% (5.9%) at 12 months after SADI and 21.2% (15.2%) and 14% (7.5%), respectively, after SADI-S. The mean (SD) preoperative body mass index was 42 (5.5) kg/m2 in the primary SADI-S group and 42.4 (9.3) kg/m2 in the revisional SADI group, and the mean (SD) follow-up was 10.1 (3.4) months. Full remission of T2D and hypertension was achieved in all patients within 6 months of surgery. There were no major complications, except for 1 case of intraoperative conversion to one-anastomosis gastric bypass.

**CONCLUSIONS:**

SADI-S is associated with significant weight loss and comorbidity resolution with a low complication rate, though larger studies are needed for further validation of these results.

## INTRODUCTION

According to the latest data, the obesity epidemic continues to grow. Over the past 50 years, the number of individuals with obesity has tripled.^1^** **Consequently, the prevalence of obesity-related conditions, including cardiovascular diseases, nonalcoholic fatty liver disease, cancer, and type 2 diabetes (T2D), is also on the rise.^2^ Currently, more than 60% of Europeans are classified as overweight or obese. In Poland, the obesity rate among adults exceeds 25%. These statistics emphasize the need to explore increasingly effective treatment approaches.^1^^;^^2^^;^^3^^;^^4^ Data show that among the operations accepted by the International Federation for the Surgery of Obesity and Metabolic Disorders, single-anas-tomosis duodeno-ileal bypass with sleeve gas-trectomy (SADI-S) is one of the most effective in terms of weight loss in both early and long-term follow-up.^5^^;^^6^^;^^7^ It can also be performed as a revisional procedure after sleeve gastrectomy (SG), in which case it involves duodenal transection with single-anastomosis-duodeno ileal bypass creation (SADI). In Poland, SG is still the option of choice in multiple bariatric centers.^8^^;^^9^The available global data indicate that within 15 years of primary SG, up to 50% of patients in certain cohorts may necessitate surgical conversion due to gastroesophageal reflux, resurgence of obesity, or comorbidities. This substantial conversion rate underscores the importance of developing more effective primary surgical approaches and provision of effective second-stage surgeries for patients.^10^^;^^11^^;^^12^** **Currently, primary SADI-S is performed in a small group of bariatric patients. This study summarizes early experiences with and results of SADI-S. To our best knowledge, this is the first Polish publication describing primary SADI-S.^13^

## AIM

 This study aims to present the results of the first group of patients treated with SADI-S or SADI in Poland.

## MATERIALS AND METHODS

 This retrospective, single-center cohort study analyzed outcomes of patients who underwent SADI-S or SADI for obesity between February 2023 and March 2024. All procedures were performed by the same surgeon. The patients were qualified for surgery according to national and international criteria.^14^ For the purposes of this publication, the abbreviation SADI was used to refer to the surgical conversion of SG to SADI-S.

Postoperative evaluation was performed during in-person follow-up visits 6 and 12 months after the surgery. It included assessment of body mass index (BMI) and weight loss reported as a percentage of total weight loss (%TWL). The patients were also asked about the presence / remission of T2D and hypertension. All outcomes were reported according the American Society for Metabolic and Bariatric Surgery (ASMBS) standards.^15^ Remission of T2D was defined as normal values of glucose metabolism indices (glycated hemoglobin A1c <6%, fasting blood glucose <100 mg/dl) in the absence of antidiabetic medications. Remission of hypertension was defined as being normotensive (blood pressure <120/80 mm Hg) without antihypertensive medications. We also analyzed the length of hospital stay, operative time, and complications throughout the follow-up. The patients who did not attend subsequent follow-up visits or did not agree to participate in the study were considered lost to follow-up and were not included in the analysis.

### Surgical technique

 All patients underwent SADI-S or SADI according to the standard technique.^5^^;^^16^ All surgeries were conducted laparoscopically in the beach chair position. The pneumoperitoneum was established using a 12-mm optical trocar placed supraumbilically. Additionally, a 12-mm port was inserted in the right and left mid-abdomen, and three 5-mm ports were inserted, one in the middle epigastrium, one in the left subcostal area, and one in the middle hypogastrium. In the initial stage of the operation, a high-energy device was utilized to skeletonize the greater curvature of the stomach, dissect the antrum approximately 3 cm from the duodenum, and cut the right gastroepiploic vessels. At the end of this stage, a retroduodenal tunnel was created. SG was performed using staplers tailored to the tissue thickness. The resection was carried out approximately 5 cm from the pylorus and 1 cm from the angle of His. The duodenum was cut using a stapler, and particular care was taken not to damage the pylorus, gallbladder, or the structures of the liver hilum. The staple line on the stomach and duodenum walls was not reinforced with sutures or clips. During the first 4 procedures, the right gastric artery remained intact, but for the subsequent surgeries, the strategy was modified and the artery was cut. Subsequently, 275 cm of the ileal loop were measured from the ileocecal valve, and a manual duodenojejunal anastomosis was performed using barbed sutures. After the anastomosis was completed, a bougie was passed through it, and a leak test was performed using methylene blue. Trocars and pneumoperitoneum were removed under visual control, with particular attention paid to bleeding. No additional sutures were used to close the fascia.

The perioperative care was guided by the Enhanced Recovery After Surgery protocol, and no drains were used. Mobilization was attempted within 2 hours of the surgery. An oral liquid diet was followed on the day of the surgery. The patients were discharged on the first postoperative day. Mechanical compression of the lower limbs was used during the procedure. Pharmacologic antithrombotic prophylaxis with a 40-mg dose of enoxaparin once daily was administered during hospitalization and continued for 10 days after discharge. The patients were recommended to use esomeprazole 40 mg once a day for 3 months and ursodeoxycholic acid 600 mg once daily for 6 months. Indocyanine green and a camera with infrared detection were used to confirm proper blood supply to the duodenal stump. Normal blood flow was confirmed in all patients, regardless of whether the gastric artery was cut or left intact.

### Statistical analysis 

The Statistica software (Statistica Inc., Kraków, Poland) was employed for statistical analysis. Descriptive statistics were used. The mean (SD) values were estimated for continuous data, whereas categorical data were presented as numbers and percentages.

### Ethics

The data were completely anonymized. The study followed international regulations on observational studies as well as the 1964 Declaration of Helsinki on ethical principles for medical research involving human subjects, including research on identifiable human material and data. Informed consent was obtained from all participants. The study protocol was approved by a bioethics committee (KB-32/24).

**TABLE 1:** Surgical outcomes

Parameter		Primary SADI-S (n = 9)	Revisional SADI (n = 3)
Sex, n (%)	Men	2 (22.2)	1 (33.3)
	Women	7 (77.8)	2 (66.7)
Age, y, mean (SD)		41.4 (9.2)	37.3 (6.5)
Preoperative BMI, kg/m2, mean (SD)		42 (5.5)	42.4 (9.3)
Length of hospital stay, d, mean (SD)		2.1 (0.3)	2 (0)
Operative time, min, mean (SD)		115 (34.2)	93 (4.7)
T2D, n (%)		7 (77.8)	2 (66.7)
Hypertension, n (%)		2 (22.2)	0

Abbreviations: BMI, body mass index; SADI, single-anastomosis duodeno-ileal bypass; SADI-S, single-anastomosis duodeno-ileal bypass with sleeve gastrectomy; T2D, type 2 diabetes

**TABLE 2:** Surgical outcomes

Parameter	Primary SADI-S		Revisional SADI	
	After 6 months (n = 9)	After 12 months (n = 3)	After 6 months (n = 3)	After 12 months (n = 2)
BMI, kg/m2, mean (SD)	30.2 (4.8)	29.8 (2.7)	32 (0.4)	30.7 (0)
%TWL, mean (SD)	27.9 (4.3)	31.1 (5.9)	21.2 (15.2)	14.0 (7.5)
T2D remission, n (%)	5 (100)	–	2 (100)	–
Hypertension remission, n (%)	3 (100)	–	–	–

Abbreviations: %TWL, percentage of total weight loss; others, seeTABLE 1

**FIGURE 1:**
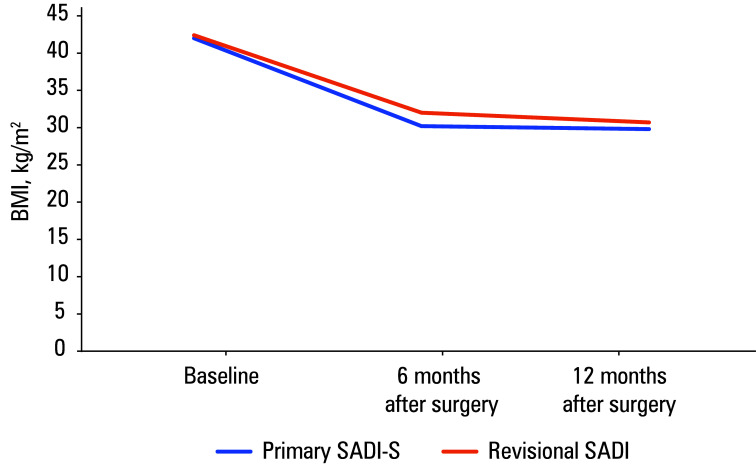
Trends in body mass index loss after primary and revisional surgery Abbreviations, seeTABLE 1

## RESULTS

A total of 12 patients were analyzed. Of those, 9 underwent primary SADI-S and 3 underwent revisional SADI. One patient (8.3%) was lost to follow-up. The patients were followed for a mean (SD) of 10.1 (3.4) months.

### Primary single-anastomosis duodeno-ileal bypass with sleeve gastrectomy

The mean (SD) age of patients undergoing primary SADI-S was 41.2 (9.2) years. The mean (SD) preoperative BMI was 42 (5.6) kg/m2, the mean (SD) length of hospital stay was 2.1 (0.3) days, and the mean (SD) operative time was 115 (34.2) minutes TABLE 1. After the surgery, the mean (SD) %TWL was 27.9% (4.3%) at 6 months and 31.1% (5.9%) at 12 months (TABLE 2,FIGURE 1). At the baseline, a total of 5 patients suffered from T2D and 3 patients had hypertension. Remission of these comorbidities was achieved in all patients within 6 months of the procedureTABLE 2.

### Revisional single-anastomosis duodeno-ileal bypass

The revisional surgeries were performed due to weight regain after primary SG in 1 patient and occurrence of T2D despite primary SG in 2 patients. The mean (SD) patient age was 37.3 (6.5) years. The mean (SD) preoperative BMI was

42.4 (9.3) kg/m2, the mean length of hospital stay was 2 days, and the mean operative time was 93.3 (4.7) minutes. The mean (SD) %TWL was 21.2% (15.2%) and 14% (7.5%), respectively, at postoperative months 6 and 12 (TABLE 2,FIGURE 1). Two patients had T2D at baseline, both of whom achieved remission 6 months after the surgeryTABLE 2.

### Complications 

There were no prolonged hospital stays or deaths in the study group. No complications occurred during the follow-up in any of the patients.

The surgery was performed in 12 of 13 qualified patients. One patient (7.9%) required intraoperative conversion to one anastomosis gastric bypass (OAGB). During the duodeno-ileal anastomosis creation, the duodenal stump tore along in the suture line. The defect was detected during the methylene blue test. The procedure was immediately converted to OAGB, as suturing of the perforation was technically impossible. The antrum was resected and a long pouch was created, as in the standard OAGB procedure. Subsequently, 150 cm of the small intestine were measured from the ligament of Treitz. A 2.5-cm-long mechanical anastomosis was made on the posterior wall of the pouch. The gastroenterotomy was closed with 2 layers of barbed continuous suture. The methylene blue test was negative. The hospital stay lasted 3 days, and the postoperative course was uncomplicated. The patient was not included in the analysis.

## DISCUSSION 

Our study shows that even in a short postoperative observation, SADI-S or SADI is associated with good weight loss results and complete resolution of comorbidities. Cutting the right gastric artery does not increase the risk of duodenal ischemia, as confirmed by an indocyanine green test. SADI-S is an advanced surgical procedure and should be performed by highly skilled bariatric and metabolic surgeons. Even major complications, such as duodenal rupture during anastomosis creation, can be effectively managed by an experienced team.

SADI- S was f i rst described by Sánchez-Pernaute et al^5^^;^^17^ as a simplified version of biliopancreatic diversion with reduced operative time and fewer surgery stages.

### Weight loss 

Available data show excellent weight loss results after both primary SADI-S and revisional SADI in the short- and long-term observation. Sánchez-Pernaute et al^5^ reported an excess weight loss of 94.7% in early follow-up. In subsequent publications, the authors confirmed the above conclusions in a medium and longterm observation.^6^^;^^18^^;^^19^** **In a study involving 91 patients undergoing SADI-S, Surve et al^20^ concluded that the mean (SD) %TWL was 34.6% (9.2%) at 1-year and 38.8% (9.9%) at 2-year follow-up. Also, Enochs et al^21^ described a %TWL of 36.8% in the second year of follow-up, which was greater than the BMI loss after Roux-en-y gastric bypass (RYGB) and laparoscopic SG. Similar results have been obtained by other authors.^22^^;^^23^

The positive effects of SADI-S on weight loss have also been demonstrated in revisional surgery. The reported %TWL 12 months after SADI ranged from 18.65% to 22%.^13^^;^^24^ Wu et al^25^ noted a %TWL of 22.34% at the 1-year follow-up; a common limb length of 200 cm was used in this study. Our results are consistent with these literature reports, even though they pertain to out- comes from the learning curve.

### Type 2 diabetes 

The available literature describes reasonable glycemic control combined with complete resolution of T2D in up to 94% of patients undergoing SADI-S, even in a short postoperative follow-up.^6^^;^^18^^;^^20^^;^^21^^;^^26^^;^^27^^;^^28^ Revisional SADI appears to be similarly effective in treating diabetes, with a remission rate of up to 94%.^13^^;^^24^^;^^25^ Moreover, Wang et al^28^ reported complete resolution of diabetes in 87% of patients with BMI below 35 kg/m2 who were initially scheduled for surgery to treat diabetes as the primary goal.

### Hypertension

Remission of hypertension after primary SADI-S occurs in 42.4% to 94% of patients.^20^^;^^29^^;^^30^ For revisional surgery, the reported remission rate is lower and ranges from 27.8% to 49.2%.^31^^;^^32^

### Major complications 

The risk of early major complications after primary SADI-S and revisional SADI is not high and amounts to 6.1%, while the reoperation rate is 3.1%. The most common complications include bleeding (1.1%), wound infection (1%), anastomotic leak (0.9%), and intra-abdominal abscess (0.6%). Duodenal stump and sleeve leakage rates are 0.3% and 0.4%, respectively. Deep vein thrombosis occurs in 0.4% of cases. Death is one of the rarest complications, occurring in 0.1% of cases.^18^^;^^22^^;^^25^;^29^^;^^30^^;^^31^^;^^33^^;^^34^^;^^35^^;^^36^^;^^37^ Although the risk of complications exceeds 6%, SADI-S should be classified as a low-risk surgery, as the rate of early complications is lower than that observed after more established procedures, such as SG and RYGB.^38^

### Limitations

The main limitation of this study is the small sample size and short follow-up period. With only 3 patients in the revisional group, it was not possible to draw definitive conclusions or make meaningful comparisons between primary and revisional procedures in terms of their effectiveness. Additionally, due to the limited number of patients, generalizability of the results is restricted. Despite these limitations, this study remains one of the first reports from Poland on the outcomes of SADI and SADI-S procedures. As such, it is valuable in the context of demonstrating the initial results of these surgeries in the Polish population.

## CONCLUSIONS 

Patients treated with SADI-S and SADI can achieve significant weight loss and high rates of T2D and hypertension remission in the early postoperative period. No complications were observed during follow-up, supporting the short-term safety of these procedures. In the case of technical difficulties, conversion to another method can be performed effectively by an experienced team. While the small sample size limits the strength of the conclusions, these findings are promising.
